# Exercise-based interventions for post-stroke depression: an overview of systematic reviews and evidence mapping

**DOI:** 10.3389/fneur.2026.1935524

**Published:** 2026-09-03

**Authors:** Songyan Zhong, Weiliang Huang, Lei Han, Beiping Li, Hui Zou, Ying Liu

**Affiliations:** 1 Zigong First People’s Hospital, Zigong, China; 2 Traditional Chinese Medicine Hospital of Renshou County, Renshou, China; 3 Department of Rehabilitation Medicine, The Second People’s Hospital of Neijiang, Neijiang, China

**Keywords:** corrected covered area, evidence map, exercise, physical activity, post-stroke depression, systematic review, umbrella review

## Abstract

**Background:**

Exercise-based interventions are increasingly used to address post-stroke depression, but existing systematic reviews differ in population definitions, intervention types, outcome measures, and methodological rigor. This overview synthesized the clinical evidence, appraised review quality and reporting, reassessed certainty, and examined primary-study overlap.

**Methods:**

Systematic reviews with or without conventional pairwise meta-analysis were eligible when they evaluated exercise-based interventions for post-stroke depression or depressive symptoms after stroke. Network meta-analyses and reviews without separately extractable exercise evidence were excluded. Methodological quality, reporting completeness, and risk of bias were assessed using AMSTAR 2, PRISMA 2020, and ROBIS, respectively. Certainty was reassessed using GRADE. Primary-study overlap was quantified using the corrected covered area and explored through sensitivity analyses.

**Results:**

Six systematic reviews were included. Two were rated low and four critically low using AMSTAR 2; ROBIS identified one review at low risk, three at unclear risk, and two at high risk. The main overlap analysis included 67 trial occurrences across 50 confirmed unique trials and indicated moderate overlap (CCA 6.80%). Most reviews reported favorable effects of exercise on depressive symptoms, although effect magnitude, heterogeneity, and certainty varied. Evidence for secondary clinical outcomes and safety was limited.

**Conclusion:**

Exercise may reduce depressive symptoms after stroke, but it remains unclear whether this finding applies specifically to patients with formally diagnosed post-stroke depression. The optimal exercise type and dose and whether benefits persist over time are also uncertain. Confidence in the evidence is limited by methodological weaknesses, heterogeneity, incomplete safety reporting, and primary-study overlap.

**Systematic review registration:**

https://www.crd.york.ac.uk/PROSPERO/view/CRD420261448674, CRD420261448674.

## 1 Introduction

Stroke remains one of the leading causes of long-term disability worldwide and is frequently accompanied by emotional and neuropsychiatric complications. Among these, post-stroke depression (PSD) is one of the most common neuropsychiatric sequelae, with pooled estimates indicating that approximately one-quarter to one-third of stroke survivors experience depression during the course of recovery (1, 2). PSD may develop at any stage after stroke, although most new-onset cases occur within the first 3 months, and a substantial proportion of affected patients experience persistent depressive symptoms over time (3). PSD is clinically important because it is associated with greater disability, poorer functional recovery, and reduced quality of life after stroke (4, 5). Observational evidence has also linked PSD to recurrent stroke and increased mortality, underscoring its adverse prognostic significance (6, 7). Despite its high prevalence and clinical consequences, PSD remains difficult to identify and manage because depressive symptoms may coexist with neurological impairment, cognitive dysfunction, communication difficulties, and fatigue, particularly during early recovery (8, 9).

Pharmacological and psychological therapies are commonly used for PSD, but the certainty of evidence supporting their effectiveness remains limited, and antidepressant treatment may be associated with central nervous system and gastrointestinal adverse events (10). These limitations have increased interest in non-pharmacological and adjunctive approaches that can be integrated into routine stroke rehabilitation. Exercise is particularly relevant because physical activity and structured exercise are established components of comprehensive stroke rehabilitation and may simultaneously address emotional symptoms, physical deconditioning, functional limitations, and reduced participation (11). Meta-analytic evidence suggests that exercise can reduce depressive symptoms after stroke (12), while broader stroke research also indicates potential benefits for health-related quality of life (13). Accordingly, a wide range of modalities has been investigated, including aerobic and resistance exercise, multicomponent rehabilitation, walking-based and home-based programmes, and mind–body approaches such as Tai Chi, Baduanjin, Qigong, and yoga (14–17).

Several systematic reviews and meta-analyses have synthesized exercise-based interventions or exercise subgroups within broader non-pharmacological treatments for depressive symptoms after stroke (12, 14, 16, 18). However, these reviews differ substantially in their clinical scope and analytical methods. Some focused on patients with established PSD, whereas others included broader stroke populations in which depressive symptoms were measured as a secondary outcome. They also differed in intervention definitions, comparators, outcome measures, eligible study designs, and analytical methods. Consequently, differences between review conclusions may reflect variation in eligibility criteria and analytical decisions rather than genuine inconsistency in the effects of exercise. Moreover, no previous overview has comprehensively compared the methodological quality, reporting completeness, risk of bias, certainty of evidence, and primary-study overlap of these reviews.

A further concern is that the same primary trials may be repeatedly included in multiple systematic reviews. Such overlap creates statistical and conceptual non-independence because several apparently separate review conclusions may be derived from the same underlying participants and trials (19, 20). Unless primary-study overlap is identified and quantified, the apparent volume and consistency of the review-level evidence may be overstated, particularly when reviews address similar populations, interventions, and outcomes (21). Therefore, this overview aimed to synthesize systematic-review evidence on exercise-based interventions for PSD and depressive symptoms after stroke.

## 2 Methods

### 2.1 Protocol and registration

This overview was conducted according to a predefined protocol and registered in the International Prospective Register of Systematic Reviews (PROSPERO; CRD420261448674). The review was reported in accordance with the Preferred Reporting Items for Systematic Reviews and Meta-Analyses 2020 statement (PRISMA 2020) (22) for overviews of systematic reviews.

### 2.2 Eligibility criteria

Systematic reviews with or without conventional pairwise meta-analysis were eligible if they evaluated exercise-based interventions for post-stroke depression or depressive symptoms after stroke. We classified the populations covered by each review as patients with formally diagnosed post-stroke depression, patients who met a prespecified cutoff on a validated depression scale, patients meeting either criterion, or broader stroke populations not selected for depression. Reviews that did not clearly report baseline depression criteria were identified separately. Reviews of broader stroke populations were eligible only when exercise-specific depression outcomes could be identified separately. These population differences were taken into account when interpreting the findings.

We defined exercise-based interventions as planned, structured programmes of physical training. Programmes delivered as part of broader rehabilitation were eligible only when the exercise component and the corresponding depression outcome could be identified separately. We grouped the interventions as aerobic or endurance exercise, resistance or strength training, walking- or circuit-based programmes, multicomponent exercise or rehabilitation combining two or more exercise components, aquatic exercise, and mind–body exercise. Mind–body exercise included yoga, Tai Chi, Baduanjin, Yijinjing, Qigong, and related traditional programmes. Comparators could include usual care, conventional rehabilitation, medication, health education, attention control, sham intervention, relaxation, no exercise, or another non-exercise intervention.

The primary outcome was depressive symptom severity assessed using a validated scale, including the Hamilton Depression Rating Scale or Hamilton Rating Scale for Depression, Geriatric Depression Scale, Beck Depression Inventory, Center for Epidemiologic Studies Depression Scale, Hospital Anxiety and Depression Scale depression subscale, Patient Health Questionnaire-9, or another validated measure. Secondary outcomes included depression response, motor or neurological function, activities of daily living, quality of life, cognitive function, sleep, anxiety, and adverse events.

Network meta-analyses were excluded because this overview focused on direct exercise-versus-comparator evidence from conventional pairwise syntheses. Network estimates may combine direct and indirect evidence and provide treatment rankings, and their interpretation depends on transitivity and consistency assumptions that would require a separate appraisal framework. Other overviews, non-systematic reviews, protocols, guidelines, conference abstracts, and primary studies were also excluded. Reviews were also excluded if they did not report a depression-related outcome or if exercise-specific evidence could not be distinguished from other non-pharmacological interventions. When multiple versions of the same review were available, the most recent or most complete version was retained.

### 2.3 Information sources and search strategy

A systematic search was conducted in PubMed/MEDLINE, Embase, the Cochrane Library, and Web of Science from database inception to May 31, 2026. The search combined controlled vocabulary and free-text terms related to stroke, post-stroke depression, depressive symptoms, exercise, physical activity, rehabilitation exercise, aerobic exercise, resistance exercise, Tai Chi, Baduanjin, Qigong, yoga, traditional Chinese exercise, systematic review, and meta-analysis. Search syntax was adapted to the requirements of each database.

The reference lists of eligible reviews and relevant overview articles were manually screened to identify additional publications.

### 2.4 Study selection and data extraction

All retrieved records were imported into a reference-management programme, and duplicate records were removed before screening. Two reviewers independently screened titles and abstracts and subsequently assessed potentially eligible reports in full text. Disagreements were resolved through discussion or consultation with a third reviewer. A primary reason for exclusion was recorded for each excluded full-text report.

Two reviewers independently extracted the following review-level information using a standardized form: first author, publication year, journal, databases searched, search end date, review type, eligible primary-study designs, number of included studies, total sample size, participant characteristics, exercise intervention, comparator, outcome measures, risk-of-bias assessment tool, and authors’ principal conclusions. Outcome-level data included the number of contributing studies and participants, effect measure, effect estimate, 95% confidence interval, heterogeneity statistic, subgroup or sensitivity analysis, publication-bias assessment, and certainty of evidence.

For reviews evaluating several non-pharmacological interventions, only separately identifiable exercise-specific evidence was extracted. When relevant information was incompletely reported in the main text, the corresponding tables, figures, reference lists, and Supplementary materials were examined. Overall review sample sizes were distinguished from outcome-specific analysis samples. Forest-plot arm totals or repeated comparison records were not interpreted as unique participants.

For the overlap analysis, primary-study identities were standardized using author name, publication year, study title, sample size, intervention, comparator, DOI, and PubMed identifier where available. Multiple publications, intervention arms, comparisons, outcomes, depression scales, and follow-up time points arising from the same underlying trial were counted as one primary trial. Records whose identities could not be resolved were excluded from the main overlap matrix rather than assigned probabilistically, because corrected covered area (CCA) (19) requires a definite trial-by-review classification and an assumed match could alter both the number of occurrences and the number of unique trials. Their possible influence was instead examined in sensitivity analyses using alternative identity assumptions.

### 2.5 Methodological and evidence appraisal

Methodological quality was assessed using A MeaSurement Tool to Assess systematic Reviews 2 (AMSTAR 2) (23). Each of the 16 items was rated as “yes,” “partial yes,” or “no.” Overall confidence was classified as high, moderate, low, or critically low according to the number of critical and non-critical weaknesses, rather than by calculating a numerical score.

Reporting completeness was evaluated using the PRISMA 2020 checklist. The 27-item checklist was operationalized as 37 main items and subitems for item-level assessment. Each item was rated as fully reported, partially reported, not reported, or not applicable.

Risk of bias in the included reviews was assessed using Risk Of Bias In Systematic reviews (ROBIS) (24). Signalling questions were evaluated across four domains: study eligibility criteria; identification and selection of studies; data collection and study appraisal; and synthesis and findings. Domain-level judgments were categorized as low, unclear, or high concern, followed by an overall judgment of low, unclear, or high risk of bias.

The certainty of evidence was reassessed by the overview authors using the Grading of Recommendations Assessment, Development and Evaluation (GRADE) approach (25) to provide a consistent framework across reviews. Evidence derived from randomized controlled trials generally started at high certainty and could be downgraded for risk of bias, inconsistency, indirectness, imprecision, or publication bias. Risk-of-bias downgrading was based on limitations in the contributing primary trials rather than on the AMSTAR 2 or ROBIS rating of the review.

Evidence combining randomized and non-randomized designs was not formally graded when the contributions of the different designs could not be separated and a defensible starting certainty could not be established. Narrative findings without a pooled estimate and safety findings without complete event denominators or comparative estimates were also not formally graded.

### 2.6 Data synthesis, evidence mapping, and overlap analysis

Because of clinical and methodological heterogeneity in exercise type, population characteristics, comparators, outcome measures, and analytical approaches, no new meta-analysis combining the included reviews was performed. Results were summarized narratively at the review and outcome levels. Effect measures, effect estimates, 95% confidence intervals, heterogeneity statistics, and directions of effect were retained as reported in the original reviews.

An evidence map was constructed to summarize the distribution of review-level evidence across depression, motor or neurological function, activities of daily living and quality of life, cognition, sleep, anxiety, and safety. Each review–outcome combination was represented once. Bubble size reflected the number of contributing primary studies, while color intensity represented the corresponding participant count when an outcome-specific denominator was available.

Primary-study overlap was quantified using the corrected covered area (19):


CCA=(N−r)/(rc−r)×100%

Where *N* represents the total number of primary-trial occurrences across reviews, *r* represents the number of unique primary trials, and *c* represents the number of contributing reviews. CCA values of 0–5%, 6–10%, 11–15%, and greater than 15% were interpreted as slight, moderate, high, and very high overlap, respectively. Classification was based on the unrounded CCA value.

The main overlap analysis included only confirmed unique exercise-specific primary trials. Outcome-specific CCA was calculated when at least two reviews contributed and trial identities could be evaluated. Sensitivity analyses examined the effects of treating unresolved records as independent trials or merging records judged likely to represent confirmed trials.

## 3 Results

### 3.1 Study selection and characteristics of included reviews

**Figure 1:**
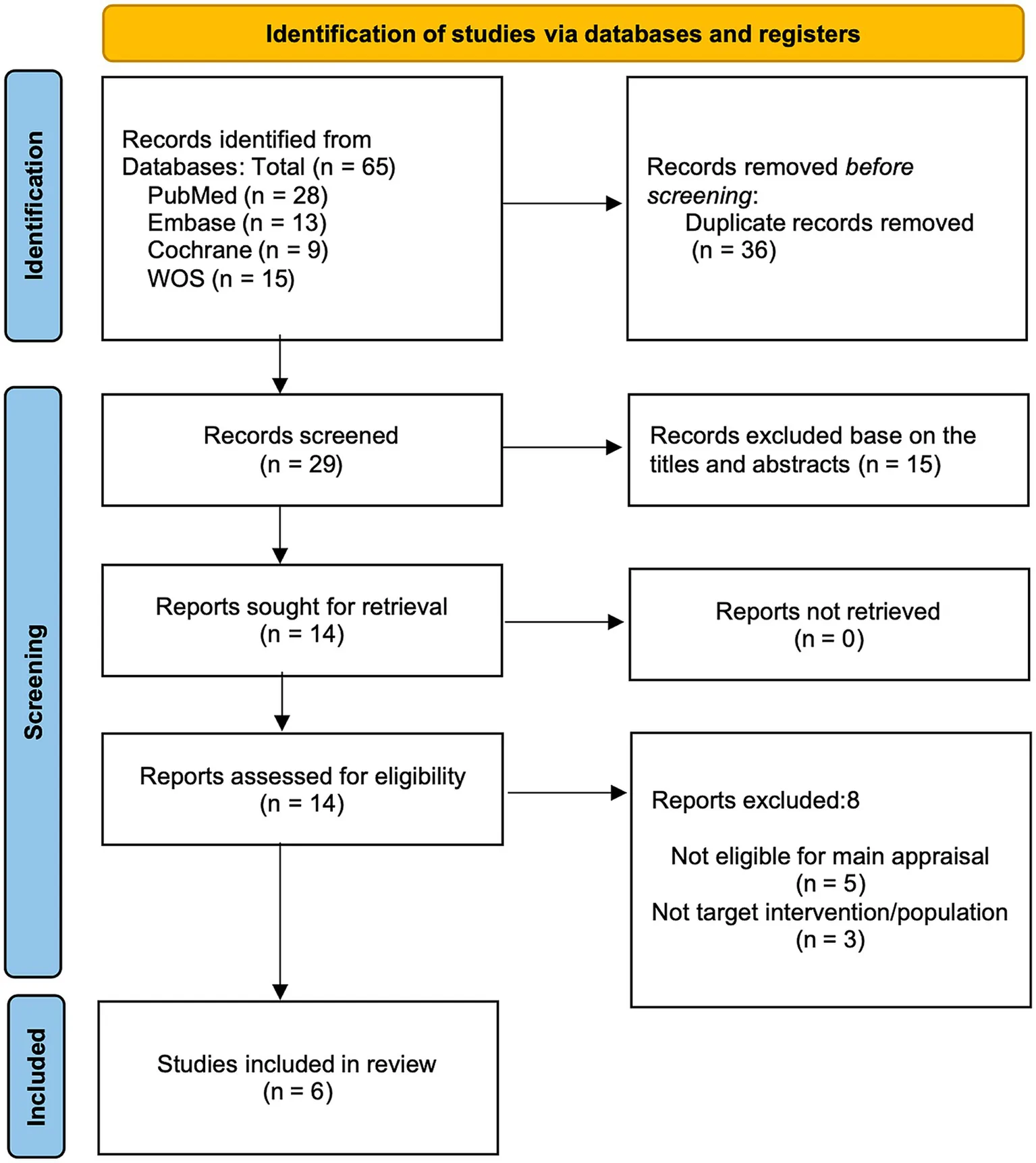
PRISMA 2020 flow diagram of study selection.

The database searches identified 65 records: 28 from PubMed, 13 from Embase, 9 from the Cochrane Library, and 15 from Web of Science. After removal of 36 duplicate records, 29 titles and abstracts were screened, of which 15 were excluded. All 14 reports sought for retrieval were obtained and assessed for eligibility. Eight reports were excluded: five did not meet the eligibility criteria for the main appraisal, and three had an ineligible intervention or population. Six systematic reviews were ultimately included in the overview. The study-selection process is shown in Figure 1.

The reviews included 10–36 primary studies and 627–2,267 participants. Five reviews primarily included randomized controlled trials (12, 16, 26–28), whereas Li 2022 (18) also included non-randomized and survey-based evidence. In Lee 2021 (16) and Shi 2025 (28), exercise represented only one component of a broader non-pharmacological evidence base.

**Figure 2:**
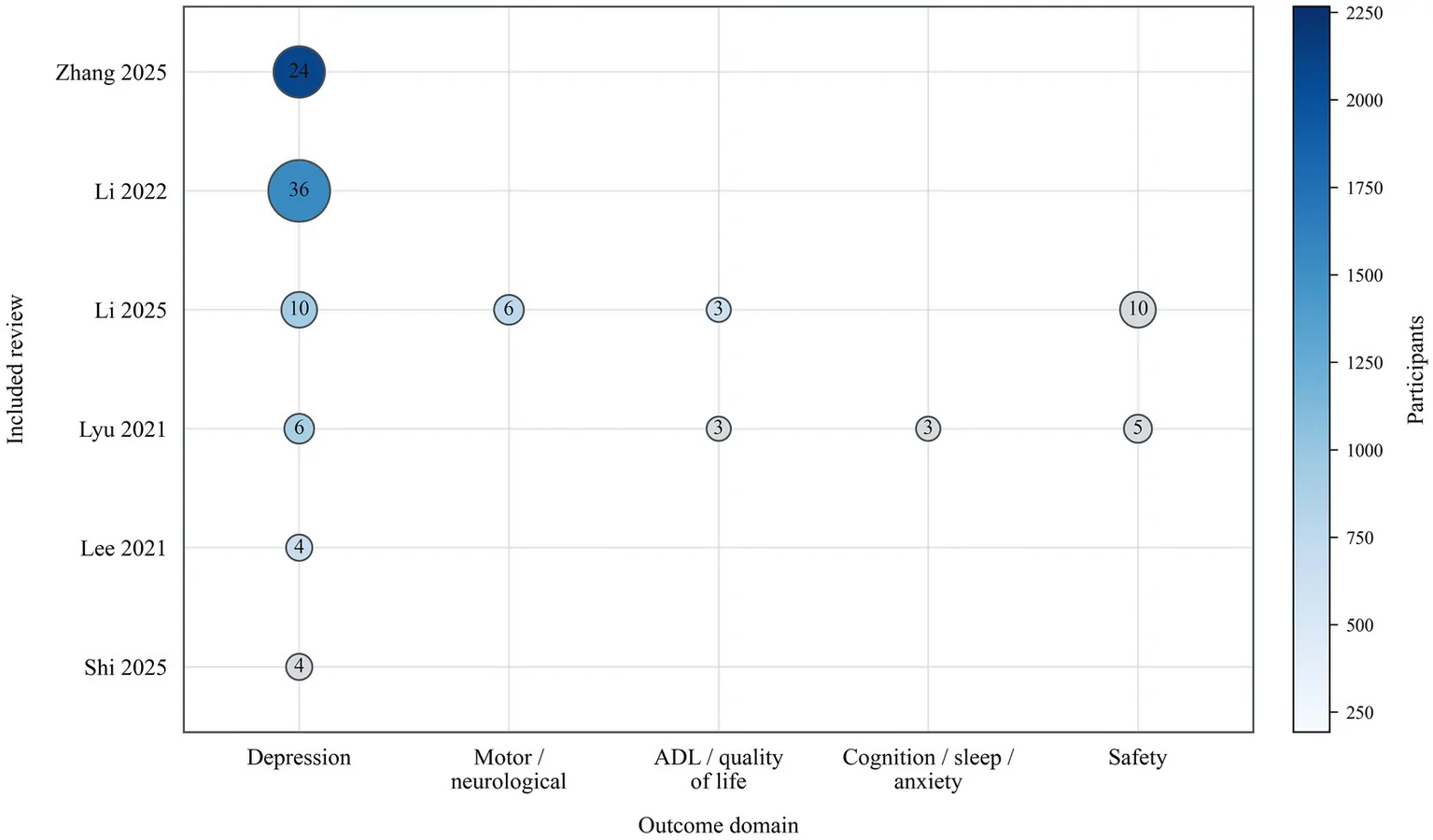
Evidence map of outcomes reported across the included systematic reviews. Bubble size represents the number of contributing studies, and color intensity represents the participant count.

The included reviews evaluated aerobic, resistance, multicomponent, rehabilitation, aquatic, home-based, traditional Chinese, and mind–body exercise. All six reviews reported depression outcomes, whereas secondary clinical outcomes and safety were less frequently assessed. Detailed characteristics are presented in Table 1, and the distribution of evidence across outcome domains is summarized in Figure 2.

**Table 1:** Characteristics of the included systematic reviews and meta-analyses.

Study	Year	Journal	Search databases	Search end date	Review type	Primary study design	No. of primary studies	No. of participants	Population	Exercise intervention	Comparator	Outcomes	Risk-of-bias tool	Main conclusion
Zhang 2025 (12)	2025	Life (Basel)	PubMed; Web of Science; Cochrane Library; Scopus; Embase	2024-09-13	SR and pairwise MA	RCTs	24	2,267	Stroke survivors; depression was the primary outcome; no baseline criterion stated	Aerobic/endurance; resistance/strength; high-intensity interval; multicomponent training; mind–body exercise (classified as aerobic in the review)	Usual care/rehabilitation/non-exercise controls	Validated depression scales	RoB 2	Exercise reduced depressive symptoms; no significant difference was found between exercise-type subgroups
Li 2022 (18)	2022	International Journal of Geriatric Psychiatry	MEDLINE; EBSCO; ScienceDirect; Web of Science; PubMed; ProQuest; CNKI; WanFang; CSTJ	Not explicitly reported	SR and pairwise MA	Experimental and survey-based empirical studies	36	1,477	Mild stroke patients; baseline PSD/depression criteria were insufficiently reported	Aerobic, resistance, and mixed aerobic-resistance exercise	Usual care, rehabilitation and other controls	Mixed depression scales	Cochrane grading system; version/items not specified	Exercise was associated with lower depressive symptoms; substantial heterogeneity remained
Li 2025 (26)	2025	Frontiers in Public Health	PubMed; Cochrane Library; Web of Science; Embase; CBM; CNKI; WanFang; VIP	2024–07	SR and pairwise MA	RCTs	10	627	Patients meeting diagnostic criteria for PSD	Mind–body/traditional Chinese exercise: Tai Chi, Baduanjin, Yijinjing, and related programmes	Routine care, medication or rehabilitation	HAMD; response; FMA; BI	Cochrane risk-of-bias tool	Traditional Chinese exercise improved HAMD and secondary functional outcomes
Lyu 2021 (27)	2021	Clinical Rehabilitation	MEDLINE; EMBASE; Cochrane Library; four Chinese databases; WHO ICTRP; ChiCTR	2020-07-27	SR and pairwise MA	RCTs	11	723	Stroke survivors; no baseline depression criterion stated	Mind–body exercise: Tai Chi	Conventional rehabilitation/usual care	Depression, mental health, sleep, cognition and anxiety	Modified 12-item assessment form based on Cochrane Handbook v5.1.0	Tai Chi improved pooled depression scores; other non-motor outcomes were uncertain
Lee 2021 (16)	2021	Topics in Stroke Rehabilitation	MEDLINE; CINAHL Plus; Scopus; Academic Search Complete; CENTRAL; LISTA	2018–08	SR and pairwise MA	RCTs	22	2090	Adults after stroke; no baseline depression criterion stated	Strength/resistance, balance, endurance/aerobic, walking-based, and robot-assisted exercise	Usual/standard treatment, sham or inactive controls	Validated depression scales	JBI-MAStARI	Exercise favored improvement numerically, but the subgroup result was not statistically significant
Shi 2025 (28)	2025	Clinical Rehabilitation	Eight English and two Chinese databases; grey literature	2025–02	SR with pairwise MA and narrative synthesis	RCTs and pilot/feasibility RCTs	17	1,297	Stroke survivors meeting diagnostic criteria or a screening cutoff for depression	Resistance/strength training; walking-based circuit training; multicomponent rehabilitation exercise with music; Yijinjing Qigong	Usual care, attention or active controls	CES-D and HAMD-24 in exercise subset	Cochrane risk-of-bias tool	Exercise-specific findings were mixed and no pooled exercise estimate was reported

BI, Barthel Index; CCA, corrected covered area; CENTRAL, Cochrane Central Register of Controlled Trials; CES-D, Center for Epidemiologic Studies Depression Scale; FMA, Fugl-Meyer Assessment; HAMD, Hamilton Depression Rating Scale; MA, meta-analysis; PSD, post-stroke depression; RCT, randomized controlled trial; SR, systematic review.

### 3.2 Methodological quality, reporting completeness, and risk of bias

According to AMSTAR 2, two reviews were rated as having low methodological quality and four as critically low. Zhang 2025 and Li 2025 (12, 26) were rated low, whereas Li 2022 (18), Lyu 2021 (27), Lee 2021 (16), and Shi 2025 (28) were rated critically low. Recurrent limitations included the absence of a complete list of excluded studies, inadequate reporting of primary-study funding, lack of a protocol or registration in some reviews, and failure to adequately assess or consider the effects of primary-study risk of bias on the synthesis and interpretation of results. Detailed item-level AMSTAR 2 judgments are provided in Table 2.

**Table 2:** AMSTAR-2 methodological quality of the included systematic reviews.

Study	PMID	D1 PICO	D2 Protocol	D3 Design	D4 Search	D5 Selection	D6 Extraction	D7 Excluded list	D8 Study detail	D9 RoB method	D10 Funding	D11 Meta methods	D12 RoB impact	D13 RoB interpretation	D14 Heterogeneity	D15 Publication bias	D16 COI	Overall	Key caveat
Zhang 2025 (12)	40003693	Y	Y	N	PY	Y	Y	N	PY	Y	N	Y	N	Y	Y	Y	Y	Low	No excluded-study citation list; no RoB-impact analysis
Li 2022 (18)	35957537	PY	N	N	PY	PY	N	N	PY	PY	N	Y	N	N	Y	Y	Y	Critically low	No protocol, duplicate extraction or excluded-study list
Li 2025 (26)	40453492	Y	Y	N	PY	Y	PY	N	Y	Y	N	Y	N	Y	Y	Y	Y	Low	No excluded-study citation list; no RoB-impact analysis
Lyu 2021 (27)	32808532	Y	Y	N	Y	Y	Y	N	Y	PY	N	Y	N	Y	PY	N	Y	Critically low	No excluded-study list; modified quality tool not fully transparent
Lee 2021 (16)	32783504	Y	N	N	Y	Y	Y	N	Y	Y	N	Y	N	Y	Y	Y	Y	Critically low	No protocol or excluded-study list; funnel-plot outlier removal
Shi 2025 (28)	40405736	Y	N	N	Y	Y	Y	N	Y	Y	N	Y	N	Y	Y	PY	Y	Critically low	No protocol/registration found; exercise subset too small for bias testing

AMSTAR-2, A MeaSurement Tool to Assess systematic Reviews 2; COI, conflict of interest; N, no; PICO, population, intervention, comparator and outcome; PY, partial yes; RoB, risk of bias; Y, yes. Overall ratings were determined from critical and non-critical weaknesses rather than a numerical score.

PRISMA 2020 reporting was incomplete and varied across reviews, with the principal gaps involving protocol-related reporting, certainty assessment, and data availability. Detailed item-level judgments are provided in Supplementary Table S1.

**Figure 3:**
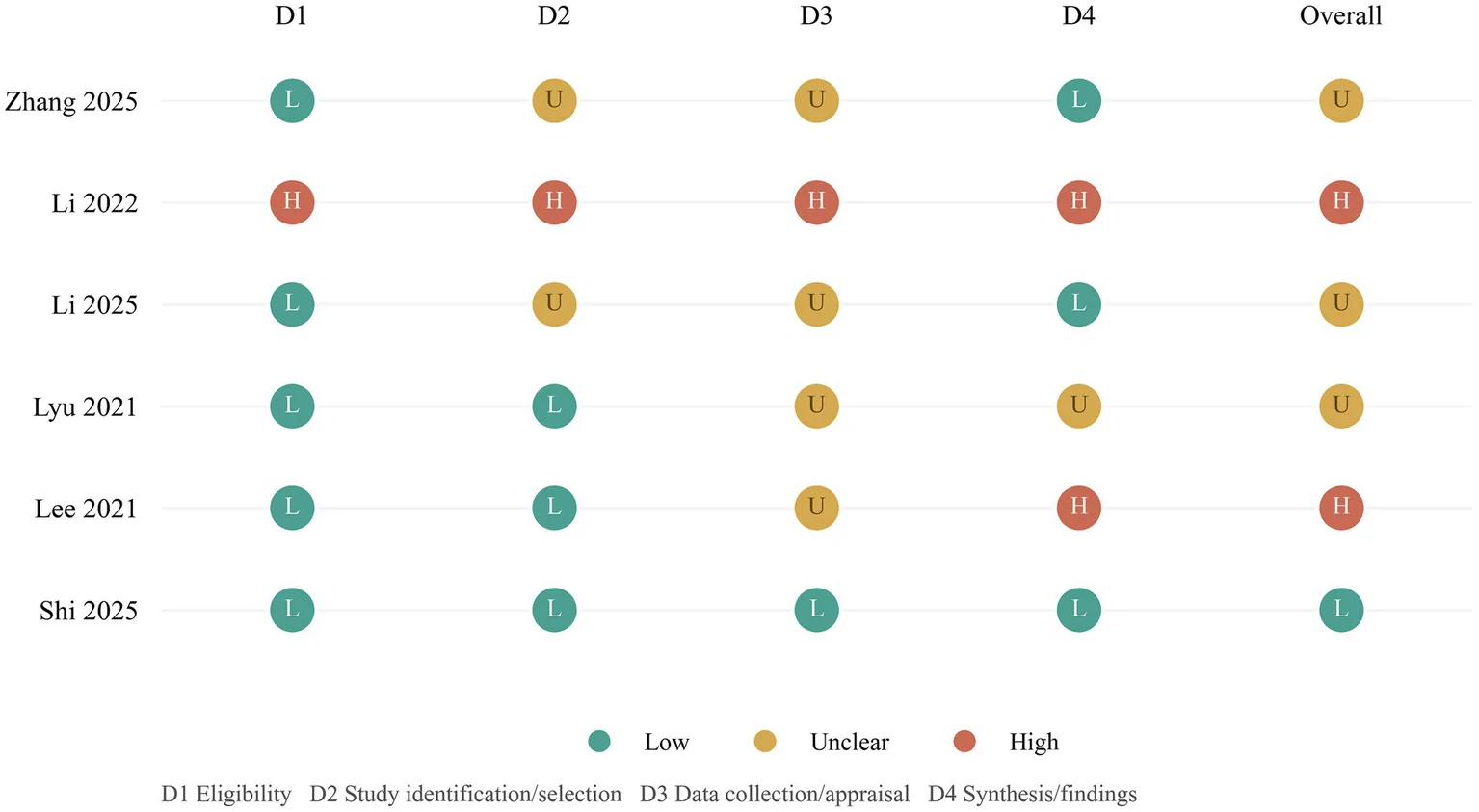
ROBIS risk-of-bias assessment of the included systematic reviews. Green, yellow, and red indicate low, unclear, and high risk of bias, respectively.

ROBIS classified one review as having low overall risk of bias, three as unclear risk, and two as high risk. In the eligibility domain, five reviews were judged to have low concern and one high concern. The principal concerns in the remaining domains related to incomplete search or selection reporting, limitations in data appraisal, and synthesis decisions that could have affected the review conclusions. Review-level judgments are presented in Table 3 and Figure 3, with the complete signalling-question assessment in Supplementary Table S3.

**Table 3:** ROBIS risk-of-bias assessment of the included systematic reviews.

Study	PMID	Domain 1 eligibility	Domain 2 selection	Domain 3 data/appraisal	Domain 4 synthesis	Overall ROBIS
Zhang 2025 (12)	40003693	Low concern	Unclear concern	Unclear concern	Low concern	Unclear risk
Li 2022 (18)	35957537	High concern	High concern	High concern	High concern	High risk
Li 2025 (26)	40453492	Low concern	Unclear concern	Unclear concern	Low concern	Unclear risk
Lyu 2021 (27)	32808532	Low concern	Low concern	Unclear concern	Unclear concern	Unclear risk
Lee 2021 (16)	32783504	Low concern	Low concern	Unclear concern	High concern	High risk
Shi 2025 (28)	40405736	Low concern	Low concern	Low concern	Low concern	Low risk

ROBIS, Risk Of Bias In Systematic reviews.

### 3.3 Primary-study overlap

**Figure 4:**
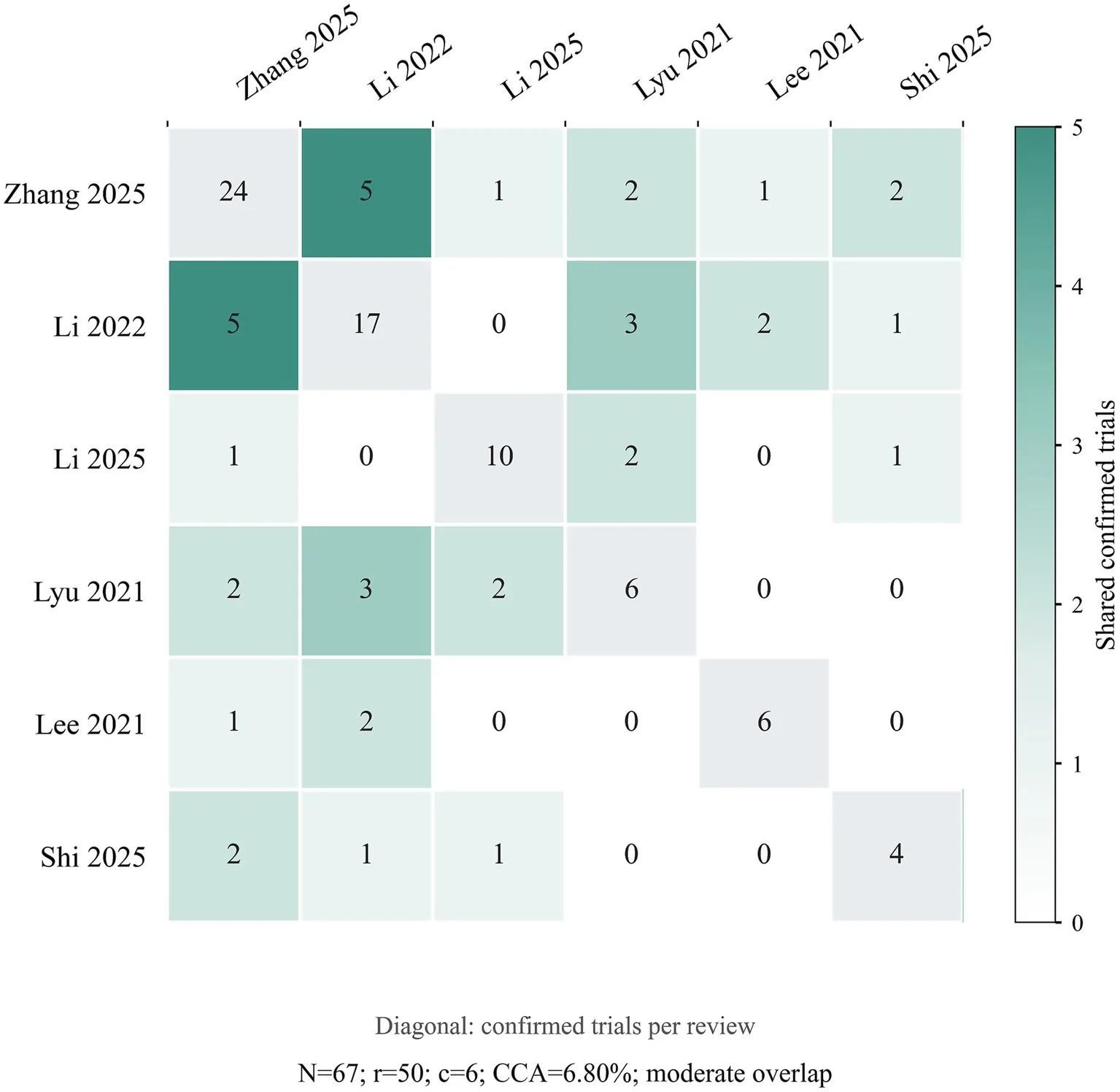
Pairwise primary-study overlap among the included systematic reviews. Darker shading indicates greater overlap.

The main overlap matrix contained 67 trial occurrences across 50 confirmed unique exercise-specific primary trials and six reviews. The overall CCA was 6.80%, indicating moderate overlap. The largest pairwise overlap occurred between Zhang 2025 (12) and Li 2022 (18), which shared five confirmed primary trials. The complete trial-by-review matrix is provided in Supplementary Table S4, and the pairwise overlap pattern is illustrated in Figure 4.

Sensitivity analyses using alternative assumptions for unresolved trial identities yielded similar CCA estimates and did not change the overall classification of moderate overlap. Detailed assumptions and results are provided in Supplementary Table S5.

Outcome-specific overlap ranged from moderate to very high, with the highest overlap observed for the Center for Epidemiologic Studies Depression Scale (CES-D). Detailed scale-specific calculations and sensitivity analyses are provided in Table 4 and Supplementary Table S5.

**Table 4:** Outcome-specific corrected covered area of primary-study overlap.

Outcome	*N*	*r*	*c*	*N* − *r*	*r* × *c* − *r*	CCA (%)	Overlap	Interpretive note
Overall depression evidence	67	50	6	17	250	6.80	Moderate	Final main analysis includes only confirmed exercise-specific unique primary trials.
HAMD/HDRS	18	13	5	5	52	9.62	Moderate	Partial verified-scale estimate; Li 2022 records with unresolved depression scales were excluded.
GDS	9	7	3	2	14	14.29	High	Conservative main estimate; if Lai/Duncan is LAI_2006, CCA = 25.00% (Very high).
CES-D	11	6	4	5	18	27.78	Very high	Working verified-scale estimate.
BDI	10	8	4	2	24	8.33	Moderate	Conservative main estimate; if the unresolved Aidar record is AIDAR_STRENGTH_2014, CCA = 14.29% (High).
HADS-D	20	17	3	3	34	8.82	Moderate	Working verified-scale estimate.
PHQ-9	1	1	1	0	0	NA	NA	Only one review contributed.
FMA	NA	NA	1	NA	NA	NA	NA	Only one review contributed.
BI/ADL	NA	NA	1	NA	NA	NA	NA	Only one review contributed.
Cognition	NA	NA	1	NA	NA	NA	NA	Only one review contributed.
Sleep	NA	NA	1	NA	NA	NA	NA	Only one review contributed.
Quality of life	NA	NA	1	NA	NA	NA	NA	Only one review contributed.

BDI, Beck Depression Inventory; BI, Barthel Index; CCA, corrected covered area; CES-D, Center for Epidemiologic Studies Depression Scale; FMA, Fugl-Meyer Assessment; GDS, Geriatric Depression Scale.

HADS-D, depression subscale of the Hospital Anxiety and Depression Scale; HAMD/HDRS, Hamilton Depression Rating Scale; NA, not applicable; PHQ-9, 9-item Patient Health Questionnaire. CCA = (*N* − *r*)/(*r* × *c* − *r*).

Where *N* is the total number of occurrences of eligible primary trials across reviews, *r* is the number of unique primary trials, and *c* is the number of contributing reviews.

### 3.4 Effects on depression

All six reviews reported exercise-related depression outcomes, although the populations, exercise interventions, scales, and synthesis methods differed. Most pooled estimates concerned outcomes measured at the end of treatment; Shi 2025 (28) also reported findings up to 1 month after treatment, whereas Li 2022 (18) did not clearly specify the assessment time. Depression was assessed using several validated clinician-rated and self-reported scales; overall and, where available, exercise-type-specific estimates are summarized in Table 5.

**Table 5:** Summary of clinical outcomes and reassessed certainty.

Study	Outcome/scale	Intervention/comparator	Assessment time	Studies/participants	Effect (95% CI)	*I* ^2^	Reassessed certainty	Main downgrade reasons	Conclusion
Zhang 2025 (12)	Depression (HADS-D, CES-D, BDI, GDS, HAMD, PHQ-9, PROMIS)	Exercise/usual care or non-exercise controls	Treatment end; duration subgroups <12 vs. ≥ 12 weeks	24 studies/2,267	SMD -0.18 (−0.30 to −0.05)	46%	Moderate	Trial-level RoB	Favors exercise
Zhang 2025 (12)	Depression: aerobic/endurance subgroup (mixed validated scales)	Aerobic/endurance exercise/controls	Treatment end; duration subgroups <12 vs. ≥ 12 weeks	11 comparisons/472	SMD -0.18 (−0.37 to 0.02)	8%	Not separately graded	Exploratory subgroup	No clear difference
Zhang 2025 (12)	Depression: resistance subgroup (mixed validated scales)	Resistance/strength exercise/controls	Treatment end; duration subgroups <12 vs. ≥ 12 weeks	2 studies/68	SMD -0.01 (−0.63 to 0.60)	36%	Not separately graded	Exploratory subgroup	No clear difference
Zhang 2025 (12)	Depression: multicomponent subgroup (mixed validated scales)	Multicomponent exercise/controls	Treatment end; duration subgroups <12 vs. ≥ 12 weeks	12 comparisons/1,220	SMD -0.24 (−0.41 to −0.06)	52%	Not separately graded	Exploratory subgroup; no between-type difference	Favors subgroup; between-type *p* = 0.75
Li 2022 (18)	Depression (scales not tabulated)	Exercise training/controls	Not explicitly reported	36 records/1,477 reported	SMD -0.41 (−0.58 to −0.23)	83.17%	Not formally graded	Mixed randomized/non-randomized evidence not separable	Favors exercise
Li 2022 (18)	Depression: aerobic subgroup (scales not tabulated)	Aerobic exercise/controls	Not explicitly reported	12 records/participants not reported	SMD -0.33 (−0.62 to −0.04)	Not reported	Not formally graded	Mixed study designs; exploratory subgroup	Favors subgroup
Li 2022 (18)	Depression: resistance subgroup (scales not tabulated)	Resistance exercise/controls	Not explicitly reported	14 records/participants not reported	SMD -0.48 (−0.75 to −0.21)	Not reported	Not formally graded	Mixed study designs; exploratory subgroup	Favors subgroup
Li 2022 (18)	Depression: mixed-exercise subgroup (scales not tabulated)	Combined aerobic-resistance exercise/controls	Not explicitly reported	8 records/participants not reported	SMD -0.49 (reported) (−0.81 to 0.58 (reported))	Not reported	Not formally graded	Reported point estimate and CI were internally inconsistent	Not interpreted; between-type *p* = 0.68
Li 2025 (26)	HAMD-17/HAMD-24	Traditional Chinese exercise/routine care, medication or rehabilitation	Treatment end; interventions 3 weeks to 3 months	10 RCTs/619	SMD -1.40 (−1.88 to −0.92)	86%	Very low	Trial-level RoB; inconsistency; Egger *p* < 0.05	Favors intervention
Li 2025 (26)	HAMD: Tai Chi subgroup	Tai Chi/controls	Treatment end; interventions 3 weeks to 3 months	5 RCTs/329	SMD -1.11 (−1.70 to −0.51)	84%	Not separately graded	Exploratory subgroup; high heterogeneity	Favors subgroup
Li 2025 (26)	HAMD: Baduanjin subgroup	Baduanjin/controls	Treatment end; interventions 3 weeks to 3 months	2 RCTs/120	SMD -1.71 (−3.42 to 0.00)	93%	Not separately graded	Exploratory subgroup; high heterogeneity	Borderline estimate
Li 2025 (26)	HAMD: other traditional exercise subgroup	Yijinjing/other traditional exercise/controls	Treatment end; interventions 3 weeks to 3 months	3 RCTs/170	SMD -1.72 (−2.79 to −0.66)	88%	Not separately graded	Exploratory subgroup; high heterogeneity	Favors subgroup; between-type *p* = 0.54
Li 2025 (26)	Overall efficacy rate	Traditional Chinese exercise/controls	Not separately reported	3 RCTs/270	OR 3.74 (1.69–8.28)	0%	Low	Trial-level RoB; imprecision	Favors intervention
Lyu 2021 (27)	Depression (HAMD, BDI, CES-D)	Tai Chi/conventional rehabilitation	12 weeks	6 RCTs/558	SMD 0.36 (0.10–0.61)	47%	Very low	Trial-level RoB; population indirectness; imprecision	Favors Tai Chi
Lee 2021 (16)	Depression: exercise subgroup (mixed validated scales)	Mixed exercise programmes/usual or standard treatment	Post-intervention	4 RCTs/263	SMD -0.24 (−0.48 to 0.01)	36%	Low	Trial-level RoB; imprecision	Numerically favors exercise; no type-specific estimate
Shi 2025 (28)	Exercise-specific depression (CES-D, HAMD-24)	Resistance, circuit, multicomponent exercise with music, and Yijinjing/controls	Post-intervention to 1 month	4 studies/not separately reported	Narrative synthesis	Not applicable	Not formally graded	No pooled exercise-specific estimate	Mixed findings; no pooled type-specific estimate
Li 2025 (26)	FMA	Traditional Chinese exercise/controls	Not separately reported	6 RCTs/373	MD 6.22 (4.12–8.32)	78%	Low	Trial-level RoB; inconsistency	Favors intervention
Li 2025 (26)	BI	Traditional Chinese exercise/controls	Not separately reported	3 RCTs/193	MD 4.95 (2.96–6.93)	65%	Very low	Trial-level RoB; inconsistency; imprecision	Favors intervention
Lyu 2021 (27)	SF-36 mental component	Tai Chi/conventional rehabilitation	12 weeks	3 RCTs/not separately reported	MD 6.15 (−3.05 to 15.36)	Not reported	Low	Trial-level RoB; imprecision	No clear difference
Lyu 2021 (27)	Sleep (PSQI)	Tai Chi/conventional rehabilitation	12 weeks	3 RCTs/not separately reported	MD 0.33 (−1.51 to 1.81)	Not reported	Low	Trial-level RoB; imprecision	No clear difference
Lyu 2021 (27)	Cognition (mixed tests)	Tai Chi/conventional rehabilitation	12–24 weeks	3 studies/not separately reported	Narrative synthesis	Not applicable	Not formally graded	No pooled estimate	Insufficient evidence
Lyu 2021 (27)	Anxiety (mixed scales)	Tai Chi/conventional rehabilitation	Not separately reported	2 studies/not separately reported	Narrative synthesis	Not applicable	Not formally graded	No pooled estimate	Inconsistent findings
Li 2025 (26)	Safety	Traditional Chinese exercise/controls	Treatment period	Review-level report/627 total	Narrative safety report	Not applicable	Not formally graded	Incomplete event definitions and denominators	No safety concerns reported by review
Lyu 2021 (27)	Safety	Tai Chi/conventional rehabilitation	Treatment period	5 studies addressed safety	Narrative safety report	Not applicable	Not formally graded	Incomplete event data across review	No events reported in five studies

BDI, Beck Depression Inventory; BI, Barthel Index; CES-D, Center for Epidemiologic Studies Depression Scale; CI, confidence interval; FMA, Fugl-Meyer Assessment; HADS-D, Hospital Anxiety and Depression Scale depression subscale; HAMD, Hamilton Depression Rating Scale; *I*^2^, *I*-squared heterogeneity statistic; MD, mean difference; OR, odds ratio; PHQ-9, Patient Health Questionnaire-9; PSQI, Pittsburgh Sleep Quality Index; RCT, randomized controlled trial; SMD, standardized mean difference. Zhang 2025 pooled HADS-D, CES-D, BDI, GDS, HAMD, PHQ-9, and PROMIS depression scores; Li 2022 did not tabulate scale-specific information or assessment times. Lee 2021 used validated depression scales but did not provide scale-specific estimates for the exercise subgroup. Exercise-type subgroup analyses were exploratory. Zhang 2025 classified mind–body exercise within its aerobic subgroup. Li 2022 reported an internally inconsistent point estimate and 95% CI for mixed exercise; the reported values are shown but not interpreted. Clinical importance could not be determined because the included reviews did not assess pooled effects against a prespecified minimal clinically important difference, and most depression results were reported as SMDs across different scales.

Zhang 2025 (12) reported that exercise reduced depressive symptoms across 24 trials involving 2,267 participants compared with usual care or non-exercise controls. The pooled standardized mean difference (SMD) was −0.18 (95% confidence interval [CI] −0.30 to −0.05; *I*^2^ = 46%). The certainty of evidence was reassessed as moderate after downgrading for trial-level risk of bias.

Li 2022 (18) reported lower depressive symptom scores with exercise across 36 records and 1,477 reported participants (SMD = −0.41, 95% CI −0.58 to −0.23; *I*^2^ = 83.17%). The subgroup estimates were −0.33 for aerobic exercise (12 records; 95% CI −0.62 to −0.04) and −0.48 for resistance exercise (14 records; 95% CI −0.75 to −0.21). The review also included eight records of combined aerobic-resistance exercise, but its reported point estimate and confidence interval were internally inconsistent. There was no evidence of a difference between exercise types (*p* = 0.68). The evidence combined randomized and non-randomized or survey-based empirical designs, and their contributions could not be separated; it was therefore not formally graded.

Li 2025 (26) found that traditional Chinese exercise improved HAMD scores across 10 randomized controlled trials involving 619 participants (SMD = −1.40, 95% CI −1.88 to −0.92; *I*^2^ = 86%). Certainty was rated very low because of trial-level risk of bias, substantial inconsistency, and evidence suggestive of publication bias. The same review reported a higher overall efficacy rate with traditional Chinese exercise across three trials and 270 participants (OR = 3.74, 95% CI 1.69 to 8.28; *I*^2^ = 0%). This evidence was rated as low certainty because of risk of bias and imprecision.

Lyu 2021 (27) reported that Tai Chi improved depressive symptoms compared with conventional rehabilitation across six randomized trials and 558 participants (SMD = 0.36, 95% CI 0.10 to 0.61; *I*^2^ = 47%). In this analysis, positive values favored Tai Chi. Certainty was rated very low because of trial-level risk of bias, population indirectness, and imprecision.

Lee 2021 (16) reported a small numerical benefit for exercise across four randomized trials involving 263 participants, but the effect did not reach statistical significance (SMD = −0.24, 95% CI −0.48 to 0.01; *I*^2^ = 36%). The evidence was rated as low certainty because of trial-level risk of bias and imprecision.

Shi 2025 (28) identified four exercise studies but did not report an exercise-specific pooled estimate. Individual-study findings were mixed, and the narrative evidence was not formally graded.

Exercise-type findings are summarized separately in Table 5. Type-specific subgroup analyses were available in three reviews (12, 18, 26), but none found a statistically significant difference between exercise types. Lee 2021 (16) and Shi 2025 (28) included several exercise modalities, including walking-based and multicomponent programmes, but did not report type-specific pooled estimates.

### 3.5 Secondary clinical outcomes

Li 2025 (26) was the only review reporting separately extractable pooled outcomes for motor function and activities of daily living. Traditional Chinese exercise improved Fugl–Meyer Assessment (FMA) scores across six randomized controlled trials involving 373 participants (mean difference [MD] = 6.22, 95% CI 4.12 to 8.32; *I*^2^ = 78%) and Barthel Index (BI) scores across three trials involving 193 participants (MD = 4.95, 95% CI 2.96 to 6.93; *I*^2^ = 65%). Certainty was low for motor function and very low for activities of daily living.

Lyu 2021 (27) reported the only separately extractable findings for quality of life, sleep, cognition, and anxiety. Neither the 36-Item Short Form Health Survey (SF-36) mental component (three trials; MD = 6.15, 95% CI −3.05 to 15.36) nor the Pittsburgh Sleep Quality Index (PSQI; three trials; MD = 0.33, 95% CI −1.51 to 1.81) showed a clear difference. Outcome-specific participant totals were not reported for either analysis, and certainty was low.

Cognitive outcomes were reported narratively in three studies using heterogeneous assessment instruments, while anxiety was narratively reported in two studies. No pooled estimates or separately reported participant totals were available. The cognitive evidence was insufficient, and the anxiety findings were inconsistent; neither outcome was formally graded.

### 3.6 Safety

Safety reporting was limited. Li 2025 and Lyu 2021 (26, 27) reported no adverse events or safety concerns, but event definitions, counts, severity, and complete denominators were generally unavailable. The remaining reviews did not provide separately extractable exercise-specific safety data; therefore, safety outcomes were not formally graded.

## 4 Discussion

### 4.1 Principal findings

This overview found that exercise may improve depressive symptoms after stroke, but the magnitude and certainty of the reported effects varied considerably. The most credible evidence supported a small benefit, whereas larger effects for traditional Chinese and mind–body exercise were based mainly on low- or very-low-certainty evidence. Overall confidence was limited by poor methodological quality, risk of bias, heterogeneity, and repeated inclusion of the same primary trials. Evidence for secondary outcomes and safety remained sparse.

### 4.2 Interpretation of the depression findings

The overall direction of evidence suggests that exercise may improve depressive symptoms after stroke, but the available reviews do not establish the superiority of any specific exercise modality. Differences in effect size are likely to reflect both clinical and intervention-related variation. Patients enrolled soon after stroke may improve partly through natural recovery and concurrent rehabilitation, while stroke chronicity and baseline depression severity affect the amount of change that can be observed; unselected samples with low symptom levels also have less room for improvement. Exercise modality, intensity, frequency, programme length, and supervision varied considerably. Supervised or higher-dose programmes may provide greater treatment exposure, whereas outcomes from less supervised programmes depend more heavily on adherence. Comparator choice can further alter the apparent effect, with smaller between-group differences expected against active rehabilitation, medication, or attention controls than against usual care or no exercise. The reviews also combined clinician-rated and self-reported depression scales and assessed outcomes at different times; SMDs allow these scores to be pooled but do not remove the underlying clinical differences. Exploratory analyses in Zhang 2025 (12), Li 2022 (18), and Li 2025 (26) did not consistently explain the remaining heterogeneity. A meta-analysis of mind–body exercise reported favorable effects on depression and functional outcomes but also identified substantial heterogeneity and frequent limitations in allocation concealment and assessor blinding (29).

The small effect reported by Zhang 2025 (12) may partly reflect the inclusion of diverse exercise interventions and broader stroke populations in which depression was not always required at baseline. By contrast, the larger effect reported by Li 2025 (26) for traditional Chinese exercise came from 10 small trials, all published in Chinese, with substantial heterogeneity, trial-level risk of bias, and possible publication bias. The evidence was therefore regionally concentrated and may not generalize to other settings. Small-study effects or selective publication may also have inflated the pooled estimate. This finding should not be interpreted as evidence that traditional Chinese exercise is superior to conventional exercise. Whether these changes are clinically meaningful also remains uncertain. None of the included reviews compared the pooled depression effects with a prespecified minimal clinically important difference. Most estimates were expressed as SMDs across several scales, and the available review-level data were insufficient to determine whether the change on any individual scale reached a meaningful threshold. Li 2025 (26) also reported a higher overall efficacy rate, but this finding was based on only three small trials and low-certainty evidence. Statistical significance alone should therefore not be taken as evidence of a clinically meaningful benefit.

Lyu 2021 (27) also reported a favorable effect of Tai Chi on depressive symptoms, but the certainty of evidence was very low. The review included broader stroke populations rather than only patients with confirmed post-stroke depression, which reduced the directness of the evidence. Tai Chi and related mind–body exercises combine physical movement with breathing control, attentional focus, relaxation, and balance practice. Their effects may therefore reflect several interacting physical and psychological components rather than exercise intensity alone. A systematic review of Tai Chi- and yoga-based interventions after stroke included only eight studies (30), most of which were small pilot or feasibility trials, and reported few clear between-group effects on psychological outcomes.

Lee 2021 (16) reported a small, non-significant effect based on four trials involving 263 participants (SMD = −0.24, 95% CI −0.48 to 0.01). Because the analysis included few trials and the confidence interval crossed the null, the result remains uncertain. Shi 2025 (28) did not provide an exercise-specific pooled estimate, and the individual findings were mixed.

### 4.3 Potential mechanisms

Exercise may alleviate depressive symptoms after stroke through neuroplastic and neurotrophic pathways. Aerobic and task-oriented exercise may modify cortical excitability and functional brain networks and promote signalling involving brain-derived neurotrophic factor, insulin-like growth factor 1, and vascular endothelial growth factor (31, 32). A post-stroke review found that evidence for exercise-related changes in brain-derived neurotrophic factor was largely preclinical and that human findings were limited and inconsistent (33). Broader depression literature supports a role for this factor in neuronal survival, synaptic plasticity, neurogenesis, and functional reorganization (34). Exercise may also plausibly influence neuroinflammation, oxidative stress, neuroendocrine regulation, monoaminergic signalling, and neurotrophic support, although some of this evidence is indirect rather than specific to post-stroke depression (35–37).

Functional and psychosocial changes may provide complementary pathways. Improvements in walking, balance, disability, and independence may increase participation and perceived control (38). Systematic reviews associate greater self-efficacy with better mobility, activities of daily living, quality of life, and fewer depressive symptoms (39, 40), while participation self-efficacy is associated with social participation (41). Supervised or group-based programmes may also provide structure, encouragement, and social support (42–44). Mind–body exercise combines these features with coordinated movement, breathing, relaxation, and attentional regulation (17, 45), although mediation by physical function, self-efficacy, or social participation has not been established.

### 4.4 Methodological quality, certainty of evidence, and primary-study overlap

The methodological quality of the included reviews was generally limited. Common weaknesses included incomplete protocol reporting, failure to provide complete lists of excluded studies, insufficient reporting of primary-study funding, and inadequate consideration of trial-level risk of bias when interpreting pooled estimates. These limitations reduce confidence in the favorable conclusions because the credibility of a pooled effect depends on both the quality of the contributing trials and the transparency of the review process.

The contrast between effect magnitude and certainty was particularly notable. Some of the largest pooled effects were supported by very-low-certainty evidence, whereas the smaller effect reported in the broad general-exercise review had the highest certainty. Larger effect estimates should therefore not automatically be regarded as more credible.

Primary-study overlap further limited the independence of the available evidence. The overall CCA indicated moderate overlap, with high or very high overlap for several depression scales. Because some reviews repeatedly included the same primary trials, their favorable conclusions were not fully independent. Sensitivity analyses using alternative assumptions for unresolved trial identities produced similar estimates and did not change the overall overlap classification. However, incomplete citation details in Li 2022 (18) limited the certainty of trial matching and the precision of the overlap analysis.

### 4.5 Secondary outcomes and safety

Evidence for motor function and activities of daily living was derived mainly from Li 2025 (26). The review reported improvements in Fugl–Meyer Assessment and Barthel Index scores, but the certainty of evidence was low or very low because of trial-level risk of bias, inconsistency, and imprecision. These findings suggest that exercise may provide functional benefits in addition to changes in mood, but the evidence remains insufficient to determine whether improvements in depression are mediated by functional recovery.

Evidence for quality of life, sleep, cognition, and anxiety was sparse. Lyu 2021 (27) found no clear effect on the SF-36 mental component or sleep quality, while cognitive and anxiety outcomes were reported narratively in only a small number of studies. These findings are insufficient to support firm conclusions.

Safety reporting was also limited. Li 2025 and Lyu 2021 (26, 27) reported no adverse events or safety concerns, but most studies did not provide complete event definitions, denominators, severity classifications, or intervention-specific data. The absence of reported adverse events should therefore not be interpreted as evidence that exercise interventions are risk-free.

### 4.6 Clinical and research implications

Exercise may be incorporated into comprehensive rehabilitation for stroke survivors with depressive symptoms. Because current evidence does not establish the superiority of any specific modality, exercise selection should be individualized according to functional status, comorbidities, safety, feasibility, and patient preference.

Future trials should use clear post-stroke depression diagnostic criteria, report baseline depression severity and concomitant treatments, and provide reproducible descriptions of exercise dose and adherence. Standardized depression, functional, quality-of-life, and safety outcomes, together with longer follow-up, are needed. Future reviews should prospectively register protocols, separate exercise-specific evidence, avoid inappropriate pooling of different study designs, and account for primary-study overlap.

### 4.7 Strengths and limitations

Strengths of this overview included the use of complementary appraisal frameworks, outcome-level GRADE reassessment, and primary-study overlap analysis based on unique underlying trials. Sensitivity analyses were conducted for unresolved trial identities, and exercise-specific evidence was separated from broader non-pharmacological findings.

Several limitations should be acknowledged. Only six systematic reviews were included, and they differed substantially in population definitions, exercise modalities, comparators, outcome scales, and eligible study designs. Because several reviews included stroke populations that were not selected for depression, the overall findings cannot be interpreted as evidence of effectiveness specifically in patients with formally diagnosed PSD. Several secondary outcomes were reported by only one review, preventing cross-review comparison and overlap analysis. Some primary-study identities and scale assignments could not be fully resolved, although sensitivity analyses did not change the overall overlap classification. Finally, this overview relied on review-level data, did not conduct a new meta-analysis, and could not draw firm conclusions about safety because adverse-event reporting was incomplete.

## 5 Conclusion

Current evidence suggests that exercise may reduce depressive symptoms after stroke, but confidence remains limited because of methodological weaknesses, clinical heterogeneity, low certainty, and primary-study overlap. It is unclear whether this finding applies specifically to patients with formally diagnosed PSD. The optimal type and dose of exercise and whether benefits persist over time are also uncertain. Exercise may nevertheless be considered as part of comprehensive stroke rehabilitation, with programme choice guided by functional status, safety, feasibility, and patient preference.

## Data Availability

The original contributions presented in the study are included in the article/Supplementary material, further inquiries can be directed to the corresponding author.
