# Supplementary material for: Exercise-based interventions for post-stroke depression: an overview of systematic reviews and evidence mapping
Source: Front Neurol. 2026 Sep 3;17:1935524. doi: 10.3389/fneur.2026.1935524 (PMC13581575; doi:10.3389/fneur.2026.1935524)
Supplement: Supplementary file 1 [file Table_1.DOCX]

**Supplementary Table S1. PRISMA 2020 item-level reporting assessment.**

| **PRISMA item** | **Item description** | **Zhang 2025** | **Li 2022** | **Li 2025** | **Lyu 2021** | **Lee 2021** | **Shi 2025** |
| --- | --- | --- | --- | --- | --- | --- | --- |
| 1 | Title | Y | Y | Y | Y | Y | Y |
| 2 | Abstract | PY | PY | PY | PY | PY | PY |
| 3 | Rationale | Y | Y | Y | Y | Y | Y |
| 4 | Objectives | Y | Y | Y | Y | Y | Y |
| 5 | Eligibility criteria | Y | Y | Y | Y | Y | Y |
| 6 | Information sources | Y | Y | Y | Y | Y | Y |
| 7 | Search strategy | PY | PY | Y | Y | Y | Y |
| 8 | Selection process | Y | Y | Y | Y | Y | Y |
| 9 | Data collection process | Y | PY | Y | Y | Y | Y |
| 10a | Data items | Y | Y | Y | Y | Y | Y |
| 10b | Other variables and assumptions | PY | Y | PY | PY | PY | PY |
| 11 | Risk of bias assessment | Y | PY | Y | Y | Y | Y |
| 12 | Effect measures | Y | Y | Y | Y | Y | Y |
| 13a | Synthesis eligibility | Y | Y | Y | Y | Y | Y |
| 13b | Data preparation | Y | Y | Y | Y | Y | Y |
| 13c | Presentation methods | Y | Y | Y | Y | Y | Y |
| 13d | Synthesis methods | Y | Y | Y | Y | Y | Y |
| 13e | Heterogeneity assessment | Y | Y | Y | Y | Y | Y |
| 13f | Sensitivity analyses | Y | N | Y | Y | N | N |
| 14 | Reporting bias assessment | Y | Y | Y | PY | Y | N |
| 15 | Certainty assessment | N | N | N | Y | N | Y |
| 16 | Study selection results | Y | Y | Y | Y | Y | Y |
| 17 | Study characteristics | Y | Y | Y | Y | Y | Y |
| 18 | Risk of bias results | Y | PY | Y | Y | Y | Y |
| 19 | Individual study results | Y | Y | Y | Y | Y | Y |
| 20a | Synthesis results | Y | Y | Y | Y | Y | Y |
| 20b | Heterogeneity results | Y | Y | Y | Y | Y | Y |
| 20c | Sensitivity results | Y | NA | Y | Y | NA | NA |
| 21 | Reporting biases | Y | Y | Y | PY | Y | NA |
| 22 | Certainty of evidence | N | N | N | Y | N | Y |
| 23 | Discussion | Y | Y | Y | Y | Y | Y |
| 24a | Registration | Y | N | Y | Y | N | N |
| 24b | Protocol | PY | N | PY | Y | N | N |
| 24c | Amendments | N | NA | N | N | NA | NA |
| 25 | Support | Y | Y | Y | Y | Y | Y |
| 26 | Competing interests | Y | Y | Y | Y | Y | Y |
| 27 | Data/code availability | Y | Y | Y | N | N | N |

Abbreviations: N, no; NA, not applicable; PRISMA, Preferred Reporting Items for Systematic Reviews and Meta-Analyses; PY, partial yes; Y, yes. The 27-item PRISMA 2020 checklist was operationalized as 37 main and sub-items for item-level assessment.

**Supplementary Table S2. Reassessed GRADE evidence profile.**

| **Study** | **Outcome** | **Studies/participants** | **Starting certainty** | **Risk of bias** | **Inconsistency** | **Indirectness** | **Imprecision** | **Publication bias** | **Final certainty** |
| --- | --- | --- | --- | --- | --- | --- | --- | --- | --- |
| Zhang 2025 | Depression: Validated depression scales | 24 studies; 2267 participants | High | Serious: 19/24 trials were rated moderate risk and 1 high risk; exercise blinding was generally infeasible | Not serious | Not serious | Not serious | Undetected but assessment limited | Moderate |
| Li 2022 | Depression: Mixed depression scales | 36 records; 1477 participants reported | Undetermined: mixed randomized/non-randomized evidence not separable | Not formally assessed | Not formally assessed | Not formally assessed | Not formally assessed | Undetected but assessment limited | Not formally graded |
| Li 2025 | Depression: HAMD | 10 RCTs; 619 participants | High | Serious: only 5/10 reported random-sequence methods; 9/10 did not report allocation concealment or blinding | Serious: I2=86% | Not serious | Not serious | Serious: funnel plot not fully symmetrical and Egger p<0.05 | Very low |
| Li 2025 | Depression response: Overall efficacy rate | 3 RCTs; 270 participants | High | Serious: allocation concealment/blinding largely unreported in the included RCTs | Not serious: I2=0% | Not serious | Serious: three small studies; event-based OIS not established and CI was wide | Undetected but assessment limited | Low |
| Lyu 2021 | Depression: Mixed validated depression scales | 6 RCTs; 558 participants | High | Serious: only 2/11 review trials were high quality; randomization, concealment and selective reporting were common limitations | Not serious: I2=47% | Serious: eligibility required stroke, not baseline PSD/depressive symptoms; scale standardization itself was not treated as indirectness | Serious: CI ranged from small/trivial to moderate benefit and no prespecified OIS/clinical threshold was available | Undetected but assessment limited | Very low |
| Lee 2021 | Depression: Exercise subgroup | 4 RCTs; 263 participants | High | Serious: participant blinding and allocation concealment were frequently unclear across included trials | Not serious: I2=36% | Not serious | Serious: small subgroup; CI included clinically relevant benefit and no effect | Undetected but assessment limited | Low |
| Shi 2025 | Depression: Exercise-specific narrative subset | 4 studies; participants not separately reported | Not applicable | Not formally assessed | Not formally assessed | Not formally assessed | Not formally assessed | Not formally assessed | Not formally graded |
| Li 2025 | Motor/neurological: FMA | 6 RCTs; 373 participants | High | Serious: randomization methods and allocation concealment/blinding were incompletely reported | Serious: I2=78% | Not serious | Not serious | Undetected but assessment limited | Low |
| Li 2025 | Activities of daily living: BI | 3 RCTs; 193 participants | High | Serious: allocation concealment/blinding largely unreported | Serious: I2=65% | Not serious | Serious: only 193 participants from three RCTs; OIS and MID were not established, and the CI may include effects below a clinically important threshold | Undetected but assessment limited | Very low |
| Lyu 2021 | Quality of life/mental health: SF-36 mental component | 3 RCTs; participants not separately reported | High | Serious: randomization/concealment/selective-reporting limitations in the underlying trial set | Not serious | Not serious | Serious: outcome-specific total unavailable; CI included harm, no effect and important benefit | Undetected but assessment limited | Low |
| Lyu 2021 | Cognition: Mixed cognitive tests | 3 studies; participants not separately reported | Not applicable | Not formally assessed | Not formally assessed | Not formally assessed | Not formally assessed | Not formally assessed | Not formally graded |
| Lyu 2021 | Sleep: PSQI | 3 RCTs; participants not separately reported | High | Serious: randomization/concealment/selective-reporting limitations in the underlying trial set | Not serious | Not serious | Serious: outcome-specific total unavailable; CI included benefit, no effect and harm | Undetected but assessment limited | Low |
| Lyu 2021 | Anxiety: Mixed anxiety scales | 2 studies; participants not separately reported | Not applicable | Not formally assessed | Not formally assessed | Not formally assessed | Not formally assessed | Not formally assessed | Not formally graded |
| Li 2025 | Safety: Adverse events | Narrative review-level report | Not applicable | Not formally assessed | Not formally assessed | Not formally assessed | Not formally assessed | Not formally assessed | Not formally graded |
| Lyu 2021 | Safety: Adverse events | 5 studies addressed safety | Not applicable | Not formally assessed | Not formally assessed | Not formally assessed | Not formally assessed | Not formally assessed | Not formally graded |

Abbreviations: BI, Barthel Index; FMA, Fugl-Meyer Assessment; GRADE, Grading of Recommendations Assessment, Development and Evaluation; HAMD, Hamilton Depression Rating Scale; MID, minimal important difference; OIS, optimal information size; PSD, post-stroke depression; RCT, randomized controlled trial; RoB, risk of bias; SF-36, 36-Item Short Form Health Survey.

**Supplementary Table S3. ROBIS signalling-question assessment.**

| **Study** | **Domain** | **Signalling question number** | **Judgment** | **Domain judgment** | **Overall ROBIS** |
| --- | --- | --- | --- | --- | --- |
| Zhang 2025 | D1 | 1.1 | Y | Low concern | Unclear risk |
| Zhang 2025 | D1 | 1.2 | Y | Low concern | Unclear risk |
| Zhang 2025 | D1 | 1.3 | Y | Low concern | Unclear risk |
| Zhang 2025 | D1 | 1.4 | Y | Low concern | Unclear risk |
| Zhang 2025 | D2 | 2.1 | Y | Unclear concern | Unclear risk |
| Zhang 2025 | D2 | 2.2 | PY | Unclear concern | Unclear risk |
| Zhang 2025 | D2 | 2.3 | PY | Unclear concern | Unclear risk |
| Zhang 2025 | D2 | 2.4 | Y | Unclear concern | Unclear risk |
| Zhang 2025 | D2 | 2.5 | Y | Unclear concern | Unclear risk |
| Zhang 2025 | D3 | 3.1 | Y | Unclear concern | Unclear risk |
| Zhang 2025 | D3 | 3.2 | Y | Unclear concern | Unclear risk |
| Zhang 2025 | D3 | 3.3 | Y | Unclear concern | Unclear risk |
| Zhang 2025 | D3 | 3.4 | Y | Unclear concern | Unclear risk |
| Zhang 2025 | D3 | 3.5 | PY | Unclear concern | Unclear risk |
| Zhang 2025 | D4 | 4.1 | Y | Low concern | Unclear risk |
| Zhang 2025 | D4 | 4.2 | Y | Low concern | Unclear risk |
| Zhang 2025 | D4 | 4.3 | Y | Low concern | Unclear risk |
| Zhang 2025 | D4 | 4.4 | Y | Low concern | Unclear risk |
| Zhang 2025 | D4 | 4.5 | Y | Low concern | Unclear risk |
| Zhang 2025 | D4 | 4.6 | Y | Low concern | Unclear risk |
| Li 2022 | D1 | 1.1 | N | High concern | High risk |
| Li 2022 | D1 | 1.2 | N | High concern | High risk |
| Li 2022 | D1 | 1.3 | PY | High concern | High risk |
| Li 2022 | D1 | 1.4 | N | High concern | High risk |
| Li 2022 | D2 | 2.1 | PY | High concern | High risk |
| Li 2022 | D2 | 2.2 | N | High concern | High risk |
| Li 2022 | D2 | 2.3 | PY | High concern | High risk |
| Li 2022 | D2 | 2.4 | PY | High concern | High risk |
| Li 2022 | D2 | 2.5 | PY | High concern | High risk |
| Li 2022 | D3 | 3.1 | PY | High concern | High risk |
| Li 2022 | D3 | 3.2 | PY | High concern | High risk |
| Li 2022 | D3 | 3.3 | PY | High concern | High risk |
| Li 2022 | D3 | 3.4 | PY | High concern | High risk |
| Li 2022 | D3 | 3.5 | N | High concern | High risk |
| Li 2022 | D4 | 4.1 | PY | High concern | High risk |
| Li 2022 | D4 | 4.2 | Y | High concern | High risk |
| Li 2022 | D4 | 4.3 | Y | High concern | High risk |
| Li 2022 | D4 | 4.4 | N | High concern | High risk |
| Li 2022 | D4 | 4.5 | PY | High concern | High risk |
| Li 2022 | D4 | 4.6 | Y | High concern | High risk |
| Li 2025 | D1 | 1.1 | Y | Low concern | Unclear risk |
| Li 2025 | D1 | 1.2 | Y | Low concern | Unclear risk |
| Li 2025 | D1 | 1.3 | Y | Low concern | Unclear risk |
| Li 2025 | D1 | 1.4 | Y | Low concern | Unclear risk |
| Li 2025 | D2 | 2.1 | Y | Unclear concern | Unclear risk |
| Li 2025 | D2 | 2.2 | PY | Unclear concern | Unclear risk |
| Li 2025 | D2 | 2.3 | PY | Unclear concern | Unclear risk |
| Li 2025 | D2 | 2.4 | Y | Unclear concern | Unclear risk |
| Li 2025 | D2 | 2.5 | Y | Unclear concern | Unclear risk |
| Li 2025 | D3 | 3.1 | Y | Unclear concern | Unclear risk |
| Li 2025 | D3 | 3.2 | Y | Unclear concern | Unclear risk |
| Li 2025 | D3 | 3.3 | Y | Unclear concern | Unclear risk |
| Li 2025 | D3 | 3.4 | Y | Unclear concern | Unclear risk |
| Li 2025 | D3 | 3.5 | PY | Unclear concern | Unclear risk |
| Li 2025 | D4 | 4.1 | Y | Low concern | Unclear risk |
| Li 2025 | D4 | 4.2 | Y | Low concern | Unclear risk |
| Li 2025 | D4 | 4.3 | Y | Low concern | Unclear risk |
| Li 2025 | D4 | 4.4 | Y | Low concern | Unclear risk |
| Li 2025 | D4 | 4.5 | Y | Low concern | Unclear risk |
| Li 2025 | D4 | 4.6 | Y | Low concern | Unclear risk |
| Lyu 2021 | D1 | 1.1 | Y | Low concern | Unclear risk |
| Lyu 2021 | D1 | 1.2 | Y | Low concern | Unclear risk |
| Lyu 2021 | D1 | 1.3 | Y | Low concern | Unclear risk |
| Lyu 2021 | D1 | 1.4 | Y | Low concern | Unclear risk |
| Lyu 2021 | D2 | 2.1 | Y | Low concern | Unclear risk |
| Lyu 2021 | D2 | 2.2 | Y | Low concern | Unclear risk |
| Lyu 2021 | D2 | 2.3 | Y | Low concern | Unclear risk |
| Lyu 2021 | D2 | 2.4 | Y | Low concern | Unclear risk |
| Lyu 2021 | D2 | 2.5 | Y | Low concern | Unclear risk |
| Lyu 2021 | D3 | 3.1 | Y | Unclear concern | Unclear risk |
| Lyu 2021 | D3 | 3.2 | Y | Unclear concern | Unclear risk |
| Lyu 2021 | D3 | 3.3 | Y | Unclear concern | Unclear risk |
| Lyu 2021 | D3 | 3.4 | PY | Unclear concern | Unclear risk |
| Lyu 2021 | D3 | 3.5 | PY | Unclear concern | Unclear risk |
| Lyu 2021 | D4 | 4.1 | Y | Unclear concern | Unclear risk |
| Lyu 2021 | D4 | 4.2 | Y | Unclear concern | Unclear risk |
| Lyu 2021 | D4 | 4.3 | Y | Unclear concern | Unclear risk |
| Lyu 2021 | D4 | 4.4 | PY | Unclear concern | Unclear risk |
| Lyu 2021 | D4 | 4.5 | Y | Unclear concern | Unclear risk |
| Lyu 2021 | D4 | 4.6 | PY | Unclear concern | Unclear risk |
| Lee 2021 | D1 | 1.1 | Y | Low concern | High risk |
| Lee 2021 | D1 | 1.2 | Y | Low concern | High risk |
| Lee 2021 | D1 | 1.3 | Y | Low concern | High risk |
| Lee 2021 | D1 | 1.4 | Y | Low concern | High risk |
| Lee 2021 | D2 | 2.1 | Y | Low concern | High risk |
| Lee 2021 | D2 | 2.2 | Y | Low concern | High risk |
| Lee 2021 | D2 | 2.3 | Y | Low concern | High risk |
| Lee 2021 | D2 | 2.4 | Y | Low concern | High risk |
| Lee 2021 | D2 | 2.5 | Y | Low concern | High risk |
| Lee 2021 | D3 | 3.1 | Y | Unclear concern | High risk |
| Lee 2021 | D3 | 3.2 | Y | Unclear concern | High risk |
| Lee 2021 | D3 | 3.3 | Y | Unclear concern | High risk |
| Lee 2021 | D3 | 3.4 | Y | Unclear concern | High risk |
| Lee 2021 | D3 | 3.5 | PY | Unclear concern | High risk |
| Lee 2021 | D4 | 4.1 | N | High concern | High risk |
| Lee 2021 | D4 | 4.2 | PY | High concern | High risk |
| Lee 2021 | D4 | 4.3 | PY | High concern | High risk |
| Lee 2021 | D4 | 4.4 | PY | High concern | High risk |
| Lee 2021 | D4 | 4.5 | N | High concern | High risk |
| Lee 2021 | D4 | 4.6 | N | High concern | High risk |
| Shi 2025 | D1 | 1.1 | Y | Low concern | Low risk |
| Shi 2025 | D1 | 1.2 | Y | Low concern | Low risk |
| Shi 2025 | D1 | 1.3 | Y | Low concern | Low risk |
| Shi 2025 | D1 | 1.4 | Y | Low concern | Low risk |
| Shi 2025 | D2 | 2.1 | Y | Low concern | Low risk |
| Shi 2025 | D2 | 2.2 | Y | Low concern | Low risk |
| Shi 2025 | D2 | 2.3 | Y | Low concern | Low risk |
| Shi 2025 | D2 | 2.4 | Y | Low concern | Low risk |
| Shi 2025 | D2 | 2.5 | Y | Low concern | Low risk |
| Shi 2025 | D3 | 3.1 | Y | Low concern | Low risk |
| Shi 2025 | D3 | 3.2 | Y | Low concern | Low risk |
| Shi 2025 | D3 | 3.3 | Y | Low concern | Low risk |
| Shi 2025 | D3 | 3.4 | Y | Low concern | Low risk |
| Shi 2025 | D3 | 3.5 | Y | Low concern | Low risk |
| Shi 2025 | D4 | 4.1 | Y | Low concern | Low risk |
| Shi 2025 | D4 | 4.2 | Y | Low concern | Low risk |
| Shi 2025 | D4 | 4.3 | Y | Low concern | Low risk |
| Shi 2025 | D4 | 4.4 | Y | Low concern | Low risk |
| Shi 2025 | D4 | 4.5 | Y | Low concern | Low risk |
| Shi 2025 | D4 | 4.6 | Y | Low concern | Low risk |

Abbreviations: H, high risk or concern; L, low risk or concern; N, no; PY, partial yes; ROBIS, Risk Of Bias In Systematic reviews; U, unclear risk or concern; Y, yes. Signalling-question judgments support, but do not mechanically determine, domain and overall judgments.

**Supplementary Table S4. Trial-by-review overlap matrix.**

| **Trial ID** | **Author, year** | **Zhang 2025** | **Li 2022** | **Li 2025** | **Lyu 2021** | **Lee 2021** | **Shi 2025** |
| --- | --- | --- | --- | --- | --- | --- | --- |
| AGUIAR_2020 | Aguiar, 2020 | 1 | 0 | 0 | 0 | 0 | 0 |
| AIDAR_AQUATIC_2012 | Aidar, 2012 | 1 | 0 | 0 | 0 | 0 | 0 |
| AIDAR_AQUATIC_2018 | Aidar, 2018 | 1 | 1 | 0 | 0 | 0 | 0 |
| AIDAR_STRENGTH_2014 | Aidar, 2014 | 1 | 0 | 0 | 0 | 0 | 0 |
| DU_2020 | Du, 2020 | 0 | 0 | 1 | 0 | 0 | 0 |
| FAULKNER_2015 | Faulkner, 2015 | 1 | 0 | 0 | 0 | 0 | 0 |
| GJELLESVIK_2021 | Gjellesvik, 2021 | 1 | 0 | 0 | 0 | 0 | 0 |
| GREEN_2002 | Green, 2002 | 0 | 1 | 0 | 0 | 0 | 0 |
| HARRINGTON_2010 | Harrington, 2010 | 0 | 1 | 0 | 0 | 0 | 0 |
| HOLMGREN_2010 | Holmgren, 2010 | 1 | 1 | 0 | 0 | 0 | 0 |
| IHLE_HANSEN_2019 | Ihle-Hansen, 2019 | 1 | 0 | 0 | 0 | 0 | 0 |
| IMMINK_2014 | Immink, 2014 | 1 | 0 | 0 | 0 | 0 | 0 |
| JUN_2013 | Jun, 2013 | 1 | 0 | 0 | 0 | 0 | 0 |
| LAI_2006 | Lai, 2006 | 1 | 0 | 0 | 0 | 1 | 0 |
| LAPOINTE_2023 | Lapointe, 2023 | 1 | 0 | 0 | 0 | 0 | 0 |
| LENNON_2008 | Lennon, 2008 | 0 | 1 | 0 | 0 | 0 | 0 |
| LIU_2021 | Liu, 2021 | 0 | 0 | 1 | 0 | 0 | 0 |
| LIU_2024 | Liu, 2024 | 1 | 0 | 0 | 0 | 0 | 0 |
| LI_2012 | Li, 2012 | 0 | 0 | 1 | 0 | 0 | 0 |
| LI_2018 | Li, 2018 | 0 | 0 | 1 | 1 | 0 | 0 |
| MACEK_2024 | Macek, 2024 | 1 | 0 | 0 | 0 | 0 | 0 |
| MAYO_2015 | Mayo, 2015 | 1 | 0 | 0 | 0 | 0 | 0 |
| MEAD_2007 | Mead, 2007 | 0 | 1 | 0 | 0 | 1 | 0 |
| MULDER_2024 | Mulder, 2024 | 1 | 0 | 0 | 0 | 0 | 0 |
| NINDORERA_2023 | Nindorera, 2023 | 1 | 0 | 0 | 0 | 0 | 0 |
| PARK_2015 | Park, 2015 | 0 | 0 | 0 | 0 | 0 | 1 |
| PARTRIDGE_2000 | Partridge, 2000 | 0 | 1 | 0 | 0 | 0 | 0 |
| PORT_2012 | Van de Port, 2012 | 1 | 1 | 0 | 0 | 0 | 0 |
| ROSENFELDT_2019 | Rosenfeldt, 2019 | 1 | 0 | 0 | 0 | 0 | 0 |
| ROWE_2017 | Rowe, 2017 | 0 | 0 | 0 | 0 | 1 | 0 |
| RYAN_2006 | Ryan, 2006 | 0 | 1 | 0 | 0 | 0 | 0 |
| SIMS_2009 | Sims, 2009 | 1 | 1 | 0 | 0 | 0 | 1 |
| SMITH_2008 | Smith, 2008 | 0 | 1 | 0 | 0 | 0 | 0 |
| SUN_2022 | Sun, 2022 | 1 | 0 | 1 | 0 | 0 | 1 |
| TANG_2023 | Tang, 2023 | 0 | 0 | 1 | 0 | 0 | 0 |
| TAYLOR_PILIAE_2012 | Taylor-Piliae, 2012 | 0 | 1 | 0 | 1 | 0 | 0 |
| TAYLOR_PILIAE_2014 | Taylor-Piliae, 2014 | 1 | 1 | 0 | 1 | 0 | 0 |
| TOPCUOGLU_2015 | Topcuoglu, 2015 | 0 | 0 | 0 | 0 | 1 | 0 |
| VAHLBERG_2017 | Vahlberg, 2017 | 0 | 0 | 0 | 0 | 1 | 0 |
| VLOOTHUIS_2019 | Vloothuis, 2019 | 1 | 0 | 0 | 0 | 0 | 0 |
| WADE_1992 | Wade, 1992 | 0 | 1 | 0 | 0 | 0 | 0 |
| WANG_2012_TAIJI | Wang, 2012 | 0 | 0 | 1 | 0 | 0 | 0 |
| WANG_2024 | Wang, 2024 | 0 | 0 | 1 | 0 | 0 | 0 |
| WERNER_1996 | Werner, 1996 | 0 | 1 | 0 | 0 | 0 | 0 |
| XIE_2018 | Xie, 2018 | 1 | 0 | 0 | 1 | 0 | 0 |
| ZEDLITZ_2012 | Zedlitz, 2012 | 0 | 1 | 0 | 0 | 1 | 0 |
| ZHANG_2023 | Zhang, 2023 | 0 | 0 | 1 | 0 | 0 | 0 |
| ZHAO_2017 | Zhao, 2017 | 0 | 0 | 1 | 1 | 0 | 0 |
| ZHAO_2022 | Zhao, 2022 | 0 | 0 | 0 | 0 | 0 | 1 |
| ZHENG_2015 | Zheng, 2015 | 0 | 1 | 0 | 1 | 0 | 0 |

Cells coded 1 indicate inclusion of the confirmed unique primary trial in the corresponding review; cells coded 0 indicate absence. The matrix includes 50 confirmed unique exercise-specific primary trials. Multiple comparisons, arms, outcomes, follow-up timepoints, and confirmed reports from the same trial were counted once.

**Supplementary Table S5. Unresolved trial identities and CCA sensitivity analyses.**

**Part A. Unresolved trial identities**

| **Record ID** | **Author/year** | **Review** | **Main identity issue** | **Treatment in main analysis** |
| --- | --- | --- | --- | --- |
| LI22_AIDAR_STRENGTH_UNRESOLVED | Aidar/2014 | Li 2022 | Probable same trial; exact report duplication unresolved | Excluded from main analysis; retained for sensitivity analysis |
| LI22_DU_2013 | Du/2013 | Li 2022 | Not reported in Li 2022 forest plot or reference list | Excluded from main analysis; retained for sensitivity analysis |
| LI22_LAI_DUNCAN_UNRESOLVED | Lai/Duncan/2003 | Li 2022 | Probable Lai trial; citation identity unresolved | Excluded from main analysis; retained for sensitivity analysis |
| LI22_FENG_2005 | Feng/2005 | Li 2022 | Not reported in Li 2022 forest plot or reference list | Excluded from main analysis; retained for sensitivity analysis |
| LI22_GUO_2017 | Guo/2017 | Li 2022 | Not reported in Li 2022 forest plot or reference list | Excluded from main analysis; retained for sensitivity analysis |
| LI22_HAN_2015 | Han/2015 | Li 2022 | Not reported in Li 2022 forest plot or reference list | Excluded from main analysis; retained for sensitivity analysis |
| LI22_KIM_2001 | Kim/2001 | Li 2022 | Not reported in Li 2022 forest plot or reference list | Excluded from main analysis; retained for sensitivity analysis |
| LI22_LI_2013 | Li/2013 | Li 2022 | Not reported in Li 2022 forest plot or reference list | Excluded from main analysis; retained for sensitivity analysis |
| LI22_LIN_2008 | Lin/2008 | Li 2022 | Not reported in Li 2022 forest plot or reference list | Excluded from main analysis; retained for sensitivity analysis |
| LI22_PAN_SHEN_2006 | Pan/Shen/2006 | Li 2022 | Not reported in Li 2022 forest plot or reference list | Excluded from main analysis; retained for sensitivity analysis |
| LI22_WANG_2010 | Wang/2010 | Li 2022 | Not reported in Li 2022 forest plot or reference list | Excluded from main analysis; retained for sensitivity analysis |
| LI22_WANG_2011 | Wang/2011 | Li 2022 | Not reported in Li 2022 forest plot or reference list | Excluded from main analysis; retained for sensitivity analysis |
| LI22_WANG_2012A | Wang/2012 | Li 2022 | Not reported in Li 2022 forest plot or reference list | Excluded from main analysis; retained for sensitivity analysis |
| LI22_WANG_2012B | Wang/2012 | Li 2022 | Not reported in Li 2022 forest plot or reference list | Excluded from main analysis; retained for sensitivity analysis |
| LI22_ZHANG_2012 | Zhang/2012 | Li 2022 | Not reported in Li 2022 forest plot or reference list | Excluded from main analysis; retained for sensitivity analysis |
| LI22_ZHAO_2004 | Zhao/2004 | Li 2022 | Not reported in Li 2022 forest plot or reference list | Excluded from main analysis; retained for sensitivity analysis |

**Part B. CCA sensitivity analyses**

| **Analysis** | **N** | **r** | **c** | **CCA** | **Overlap** | **Interpretation** |
| --- | --- | --- | --- | --- | --- | --- |
| Final main analysis | 67 | 50 | 6 | 6.80% | Moderate | Primary submission result |
| Sensitivity A | 83 | 66 | 6 | 5.15% | Moderate | Upper r assumption; unresolved identities not merged |
| Sensitivity B | 83 | 64 | 6 | 5.94% | Moderate | Assumes two probable identities are duplicates of confirmed trials |

Abbreviations: CCA, corrected covered area. The main analysis included confirmed unique exercise-specific primary trials only. Unresolved trial identities were excluded from the main matrix and examined only in sensitivity analyses.
